# Supplementary material for: Viewpoints of pregnant mothers and community health workers on antenatal care in Lweza village, Uganda
Source: PLoS One. 2021 Feb 16;16(2):e0246926. doi: 10.1371/journal.pone.0246926 (PMC7886125; doi:10.1371/journal.pone.0246926)
Supplement: S2 File — This is the questionnaire used with the 100 village women. (DOCX) [file pone.0246926.s002.docx]

**Household Questionnaire**

**[Read Verbal Consent Statement]**

**I agree to participate in this study.**

**Signature/Thumb**  .

(If the participant consents, proceed with the interview.)

**Background information:**

1. Gender
2. Female
3. Male
4. Age group
5. 19 and under
6. 20-24
7. 25 and older
8. Have you ever gone to school?
9. Yes
10. No

3.1 If yes, what is your highest level of education?

1. Primary
2. Secondary
3. Tertiary Institution
4. Degree
5. What is your religious affiliation?
   1. Catholic
   2. Protestant (Church of Uganda)
   3. Pentecostal
   4. Muslim
   5. Buddhist
   6. Other………………………………………………………………

**The next section of questions is going to ask you about your knowledge and concerns regarding maternal health. Please feel free to share with me what you know. There’s no right or wrong answers, and all of your responses will be highly valued. If there are any questions you wish not to answer, it’s ok and we can skip it.**

1. Have you ever been pregnant?
2. Yes
3. No

(If yes, continue to the next question. If no, stop the interview.)

1. How many times have you been pregnant?
   1. One
   2. Two
   3. Three
   4. Four
   5. Five or more
2. During your pregnancy/pregnancies, did you have access to pregnancy health education?
   1. Yes
   2. No
   3. If yes, where did you get that information? *Choose all that apply.*
3. Relative
4. Neighbors
5. Government Health Facility
6. Private Health Facility
7. Village Health Team (VHT)
8. Public Talk
9. Internet
10. Traditional Birth Attendant
11. Other
12. How old were you when you first got pregnant?
    1. 19 or under
    2. 20-24
    3. 25 or older
13. Was your first pregnancy planned?
    1. Yes
    2. No
14. How many antenatal care appointments did you attend during your first pregnancy?
    1. Less than 4
    2. 4-7
    3. 8 or more
15. Who accompanies you when you go to your antenatal appointments?
    1. Husband
    2. Relative
    3. Community member
    4. VHT
    5. None
16. Where did you deliver your first baby from, or if you are pregnant with your first baby, where do you intend to deliver your baby?
    1. Government Health Facility
    2. Private Health Facility
    3. Traditional Birth Attendant
    4. Other
17. How easy is it for you to access antenatal health education?
    1. Very easy
    2. Easy
    3. Not easy, but not hard
    4. Hard
    5. Very hard
18. During your antenatal clinic, what do you usually learn about? *Circle all that apply*
    1. Nutrition while I’m pregnant
    2. Nutrition for my baby
    3. Breastfeeding
    4. Family planning and/or pregnancy spacing
    5. Caring for my newborn
    6. Preparing for labor and delivery
    7. HIV/AIDS prevention
    8. Malaria prevention during pregnancy
    9. Post-partum depression
    10. Personal hygiene of mother during pregnancy
    11. Obstetric complications (fistulas, c-sections, etc)
    12. The importance of attending antenatal regularly
19. Some people feel depressed after delivering their babies. Have you ever experienced depression or gone through a similar experience after having a baby?
    1. Yes
    2. No (if no, skip to number 16)

15.1 If yes, what was the cause of the depression?

15.2 Did you seek help?

a. Yes

b. No

15.3 If yes, what type of help did you get?

a. Talked to relatives

b. Counseling

c. Talked to a doctor or health worker

d. Consulted a traditional healer

e. Talked to a VHT

f. Other

1. During your pregnancy, do you feel a strong connection to other pregnant women?
   1. Yes
   2. No
2. During your pregnancy, did you change your diet?
   1. Yes
   2. No
   3. If yes, who recommended a diet change?
      1. Health worker
      2. Traditional Birth Attendant
      3. Self
      4. Husband or relative
      5. Community member or friend
      6. VHT
      7. Others
3. During pregnancy, some people are recommended to take additional medicines or treatment (vitamins, herbs, etc) to help them with their pregnancy. Did you take any medicines or treatments to help you with your pregnancy?
   1. Yes
   2. No

18.1 If yes, what type of medicine or treatment did you get?

…………………………………………………………………………………………………………………………….

- 1. If yes, who recommended you to take those medicines or treatments?
     1. Health worker
     2. Traditional Birth Attendant
     3. Relative or friend
     4. VHT
     5. Self
     6. Other

**The next set of questions is asking how much you know about information concerning maternal health. It has a scale ranging from 0 to 5, where 0 means you know nothing and 5 means you know everything. A 1 means you know a little bit, a 2 means you know some things, a 3 means you know many things, and a 4 means you know most things. Please circle the most appropriate number concerning your knowledge on the following subjects.**

1. On a scale of 0 to 5, how much do you feel that you know about nutrition during pregnancy?

*0 1 2 3 4 5*

*Nothing Everything*

1. On a scale of 0 to 5, how much do you feel that you know about breastfeeding?

*0 1 2 3 4 5*

*Nothing Everything*

1. On a scale of 0 to 5, how much do you feel that you know about family planning?

*0 1 2 3 4 5*

*Nothing Everything*

1. On a scale of 0 to 5, how much do you feel that you know about caring for your newborn?

*0 1 2 3 4 5*

*Nothing Everything*

1. On a scale of 0 to 5, how much do you feel that you know about common discomforts of pregnancy?

*0 1 2 3 4 5*

*Nothing Everything*

1. On a scale of 0 to 5, how much do you feel that you know about the process of labor and delivery?

*0 1 2 3 4 5*

*Nothing Everything*

1. On a scale of 0 to 5, how much do you feel that you know about HIV transmission from mother to her newborn baby?

*0 1 2 3 4 5*

*Nothing Everything*

1. On a scale of 0 to 5, how much do you feel that you know about preventing malaria during pregnancy?

*0 1 2 3 4 5*

*Nothing Everything*

1. On a scale of 0 to 5, how much do you feel that you know about depression after delivering a baby?

*0 1 2 3 4 5*

*Nothing Everything*

1. On a scale of 0 to 5, how much do you feel that you know about obstetric complications like emergent cesarean sections or fistulas?

*0 1 2 3 4 5*

*Nothing Everything*

1. On a scale of 0 to 5, how prepared did you feel to have your first baby?

*0 1 2 3 4 5*

*Not prepared Extremely prepared*

Questions #30-40

Please rate your agreement with the following statements:

| Statement | Strongly Agree | Agree | Neutral | Disagree | Strongly Disagree |
| --- | --- | --- | --- | --- | --- |
| 30. I feel that I learned enough about nutrition during my pregnancy. |  |  |  |  |  |
| 31. I feel that I learned enough about breastfeeding during my pregnancy. |  |  |  |  |  |
| 32. I feel that I learned enough about family planning during my pregnancy. |  |  |  |  |  |
| 33. I feel that I learned how to properly care for my newborn during my pregnancy. |  |  |  |  |  |
| 34. I feel that I learned enough about the common discomforts during pregnancy. |  |  |  |  |  |
| 35. I feel that I learned enough to be adequately prepared for the labor and delivery process during my pregnancy. |  |  |  |  |  |
| 36. I learned enough about HIV prevention during my pregnancy. |  |  |  |  |  |
| 37. I learned enough about malaria prevention during my pregnancy. |  |  |  |  |  |
| 38. I learned about the signs of post-partum depression and what to do if I think I have it after my delivery. |  |  |  |  |  |
| 39. I learned about the obstetric complications like cesarean sections and fistulas and what to do if they happen to me. |  |  |  |  |  |
| 40. I felt properly prepared to have my first baby. |  |  |  |  |  |

**Thank you so much for your participation in this questionnaire. What questions do you have for us?**
