# Supplementary material for: Viewpoints of pregnant mothers and community health workers on antenatal care in Lweza village, Uganda
Source: PLoS One. 2021 Feb 16;16(2):e0246926. doi: 10.1371/journal.pone.0246926 (PMC7886125; doi:10.1371/journal.pone.0246926)
Supplement: S3 File — This guide was used to conduct the focus group discussion with both the teachers and the Village Health Project- Uganda groups. (DOCX) [file pone.0246926.s003.docx]

**Focus Group Discussion Guide**

**[Read Verbal Consent]**

**Participation in this study is voluntary. There will be a small reimbursement given at the end of the focus group discussion to thank you for taking the time to help the research team with their investigation.**

The research team has already conducted interviews with 100 women in this community. The interviews were also on the same topics of maternal health and pregnancy education. The questions and topics we will be discussing today are to find more information out about some of the interesting things that came up during those surveys. We will be moving from topic to topic, so if there’s anything you’d like to add please feel free to share with us what you know.

1. Let’s start with introductions. If you could tell us your name and how many pregnancies you’ve had, that would be a great start!
2. How many antenatal care appointments did you attend during your first pregnancy?
   1. What are some of the reasons for going or not going?
   2. How do you decide where to go for antenatal?
3. How long does a typical wait last for antenatal appointments?
   1. Government versus private…
   2. What do you do while you’re waiting?
4. What role does your husband and family play in supporting you during your pregnancy?
5. During your antenatal clinic, what things did you usually learn about regarding your pregnancy?
   1. Who taught you those things? When did this learning happen?
6. Some people feel depressed after delivering their babies. Have you ever experienced depression or know someone who has gone through a similar experience after having a baby?
   1. If yes, what are the reasons for women experience it?
7. During your pregnancy, did you change your diet?
   1. If yes, who recommended a diet change?
   2. What changes did you make?
8. Many women in your community said they take herbs to help them with their pregnancy. What herbs are people taking?
   1. What do these herbs help with?
   2. Where do you get the herbs?
   3. Who recommends the herbs?
9. During your pregnancy, what did you worry the most about related to being pregnant?
10. How prepared did you feel to have your first baby?
    1. What are some reasons for that?
11. What was the best part about the care you received during your pregnancy?
12. What was the worst part about your pregnancy?
13. We are seeing a pattern in your community that over half of women get pregnant before the age of 20.
    1. What are some of the reasons for this pattern? Do you think this needs to be changed?
    2. When do people start using family planning?
    3. Are men involved in family planning?
14. Many people feel that they don’t know enough about family planning. Could we talk a little bit about how people learn about family planning, how women make decisions to use it, and when it is typically used?
    1. Are there areas of improvement you see?
15. If a woman does not choose to breastfeed, what other options are available for her baby?
    1. Is formula affordable?
    2. What other sources of milk are used?

- Those were all of the questions and topics I had for today- is there anything else anyone would like to add or share to the focus group discussion today?
- Thank you so much for participating. If anybody has any questions, I will leave my contact information here for you to access.
