# Supplementary material for: Viewpoints of pregnant mothers and community health workers on antenatal care in Lweza village, Uganda
Source: PLoS One. 2021 Feb 16;16(2):e0246926. doi: 10.1371/journal.pone.0246926 (PMC7886125; doi:10.1371/journal.pone.0246926)
Supplement: S4 File — This interview guide was used to interview the TBA. (DOCX) [file pone.0246926.s004.docx]

**Key Informant: Traditional Birth Attendant Guide**

**[Read Verbal Consent]**

1. As a traditional birth attendant, what role do you play in a woman’s pregnancy?
2. What questions or complaints do pregnant women commonly come to you with?
3. What herbs or treatments do you commonly recommend to women to help with their pregnancy?
   1. What do these herbs and treatments help with?
   2. How do women get them?
4. What herbal treatments do you use or recommend for labor?
   1. What herbal treatments do you use or recommend for delivery/breaking?
5. What do you worry the most about, related to the health of a pregnant woman?
6. In this community, what do you think are obstacles women face that keep them from having healthy pregnancies?
7. In this community, what do you think could be improved in order to help more women achieve healthy pregnancies?
   1. *A healthy pregnancy involves good foods, medications, not falling ill, etc*

**Thank you so much, I appreciate all that you have shared with me today!**
