# Supplementary material for: Viewpoints of pregnant mothers and community health workers on antenatal care in Lweza village, Uganda
Source: PLoS One. 2021 Feb 16;16(2):e0246926. doi: 10.1371/journal.pone.0246926 (PMC7886125; doi:10.1371/journal.pone.0246926)
Supplement: S5 File — This guide was used to interview a Village Health Team member. (DOCX) [file pone.0246926.s005.docx]

**Key Informant: VHT Guide**

1. When you find out a woman is pregnant, what advice or recommendations do you give to her regarding her pregnancy?
2. What is your role, as a VHT, in a woman’s pregnancy?
   1. Do you make any dietary, medication, or treatment recommendations?
   2. If yes, where do you learn about those recommendations?
3. Are there questions or complaints that pregnant women commonly come to you with?
   1. If so, what are they?
4. What do you worry about most, related to the health of a pregnant woman?
5. In this community, what do you think are obstacles women face that keep them from having healthy pregnancies?
6. In this community, what do you could be improved in order to help more women achieve healthy pregnancies?
7. In your opinion, is teenage pregnancy a problem here? Why or why not?
   1. If it’s a problem, do you have any suggestions for how to change it?
8. Could you talk a little bit about how people learn about family planning, and some of the obstacles people face when trying to use it?

**Thank you so much, I appreciate all that you have shared with me today!**
