# Supplementary material for: Viewpoints of pregnant mothers and community health workers on antenatal care in Lweza village, Uganda
Source: PLoS One. 2021 Feb 16;16(2):e0246926. doi: 10.1371/journal.pone.0246926 (PMC7886125; doi:10.1371/journal.pone.0246926)
Supplement: S6 File — This guide was used to interview the midwives. (DOCX) [file pone.0246926.s006.docx]

**Key informant: Midwives Guide**

1. How many antenatal visits do you recommend?
   1. At what stage do you recommend an expectant mother to start attending antenatal care appointments?
   2. What happens at each of those antenatal visits?
2. At what time are the health talks conducted?
   1. Where are the health talks conducted?
3. Are there topics or standards that exist for what pregnancy education needs to be taught to women and when?
   1. If so, who sets the standards/topics?
   2. What barriers exist to delivering this health education?
4. Do you routinely recommend any dietary changes to pregnant women?
   1. What types of food do you recommend and why?
5. Do you routinely recommend any new medications or treatments (vitamins, herbal medicines, etc) to help women with their pregnancy?
   1. What are those recommendations?
6. What do you worry the most about, related to the health of a pregnant woman?
7. What do you think the hospital does well regarding antenatal care?
8. What improvements would you like to see regarding antenatal care?
9. After surveying 100 women in Lweza, we found that the majority of women first become pregnant as a teenager. Could you talk a little about this, why it might be, and what we could do to change it if you’d like to see it changed?
10. What conversations do you have with women regarding family planning?
    1. Is child spacing important?
    2. Why or why not?
    3. What family planning methods do you routinely recommend?
11. What training do you need to complete to become a certified midwife in Uganda?

**Thank you so much, I appreciate all that you have shared with me today!**
