# Supplementary material for: Viewpoints of pregnant mothers and community health workers on antenatal care in Lweza village, Uganda
Source: PLoS One. 2021 Feb 16;16(2):e0246926. doi: 10.1371/journal.pone.0246926 (PMC7886125; doi:10.1371/journal.pone.0246926)
Supplement: Attachment — Submitted filename: Response to Reviewers.docx [file pone.0246926.s007.docx]

**Ryan McAdams, M.D.**

*Neonatology Division Chief*

**Division of Neonatology**

Department of Pediatrics

UW School of Medicine & Public Health

Mail Code 4108, 600 Highland Ave

Madison, WI 53792-8370

Phone (608) 417-6236

FAX (608) 417-6562

mcadams@pediatrics.wisc.edu

January 26, 2021

Frank T. Spradley
Academic Editor
PLOS ONE

Dear PLOS ONE Editorial Team,

Thank you for considering our manuscript (PONE-D-20-05007), “Viewpoints of Pregnant Mothers and Community Health Workers on Antenatal Care in Lweza Village, Uganda,” for publication in *PLOS ONE*. We appreciate the opportunity to further revise our manuscript based on the valuable feedback from the editorial team and the reviewers. We have included a detailed, point-by-point response to the reviewers' comments (see below). We hope you agree that our manuscript is now acceptable for publication in *PLOS ONE*.

Research support was provided by an award from the University of Wisconsin School of Medicine and Public Health and the Herman and Gwendolyn Shapiro Foundation.

**Responses to the Reviewers**

**Comments to the Author**

1. If the authors have adequately addressed your comments raised in a previous round of review and you feel that this manuscript is now acceptable for publication, you may indicate that here to bypass the “Comments to the Author” section, enter your conflict of interest statement in the “Confidential to Editor” section, and submit your "Accept" recommendation.

Reviewer #1: All comments have been addressed

Response: We thank the reviewer for acknowledging that we have addressed all the reviewer’s comments.

2. Is the manuscript technically sound, and do the data support the conclusions?

The manuscript must describe a technically sound piece of scientific research with data that supports the conclusions. Experiments must have been conducted rigorously, with appropriate controls, replication, and sample sizes. The conclusions must be drawn appropriately based on the data presented.

Reviewer #1: Yes

Response: We thank the reviewer for agreeing that the manuscript is technically sound and that the data support the conclusions.

3. Has the statistical analysis been performed appropriately and rigorously?

Reviewer #1: I Don't Know

Response: As described in the Methods section, we took a standard approach to analyze quantitative data using IBM SPSS Statistics for descriptive statistics such as frequencies and percentages. We used an established thematic approach to analyze qualitative data (e.g., open-ended question responses). Therefore, we believe our statistical analysis was performed appropriately and rigorously.

 4. Have the authors made all data underlying the findings in their manuscript fully available?

The [PLOS Data policy](http://www.plosone.org/static/policies.action#sharing) requires authors to make all data underlying the findings described in their manuscript fully available without restriction, with rare exception (please refer to the Data Availability Statement in the manuscript PDF file). The data should be provided as part of the manuscript or its supporting information, or deposited to a public repository. For example, in addition to summary statistics, the data points behind means, medians and variance measures should be available. If there are restrictions on publicly sharing data—e.g. participant privacy or use of data from a third party—those must be specified.

Reviewer #1: Yes

Response: Thank you for recognizing that we have made all data underlying the findings in our manuscript fully available.

 5. Is the manuscript presented in an intelligible fashion and written in standard English?

PLOS ONE does not copyedit accepted manuscripts, so the language in submitted articles must be clear, correct, and unambiguous. Any typographical or grammatical errors should be corrected at revision, so please note any specific errors here.

Reviewer #1: Yes

Response: Thank you. We again reviewed the manuscript for any typographical or grammatical errors.

 6. Review Comments to the Author

Please use the space provided to explain your answers to the questions above. You may also include additional comments for the author, including concerns about dual publication, research ethics, or publication ethics. (Please upload your review as an attachment if it exceeds 20,000 characters)

Reviewer #1: Thank you for addressing all my suggestions

I think it reads well however if there is going to be further review I recommend:
- Ensuring that all the acronyms are entered after the first full text is provided

Response: Thank you. We have updated all acronyms so that they are entered after the first full text.

- Consider reducing the amount of acronyms

Response: Thank you. We reduced the amount of acronyms throughout the manuscript. The most common one used was for antenatal care (ANC), which appeared abundantly throughout the paper. Removal of “ANC” allows for easier reading. The acronyms “PPD” (postpartum depression) and “VHT” (Village Health Team member) were also eliminated to improve the flow of the paper.

- That your review to positioning of the additional information provided in lines 294-305 - would it fit better in another section of the paper?

Response: Thank you. The paragraph on literacy level was moved to line 281 where it fits with the demographic information about the respondents. The breastfeeding paragraph remained at line 336, where it fits in with the section about what women reported learning about.

Respectfully,

Mackenzie Delzer	Ryan McAdams, MD

Neonatology Division Chief, Department of Pediatrics

Univ of Wisconsin School of Medicine and Public Health
